# Developmental Assessments during Injury Research: Is Enrollment of Very Young Children in Crèches Associated with Better Scores?

**DOI:** 10.3390/ijerph14101130

**Published:** 2017-09-26

**Authors:** Divya Nair, Olakunle Alonge, Jena Derakhshani Hamadani, Shumona Sharmin Salam, Irteja Islam, Adnan A. Hyder

**Affiliations:** 1 Department of International Health, International Injury Research Unit, Johns Hopkins University Bloomberg School of Public Health, Baltimore, MD 21205, USA; oalonge1@jhu.edu (O.A.); ahyder1@jhu.edu (A.A.H.); 2 IDinsight, 24/1 Hauz Khas Village, New Delhi 110021, India; 3 International Centre for Diarrhoeal Disease Research, GPO Box 128, Dhaka 1000, Bangladesh; jena@icddrb.org (J.D.H.); shumona@icddrb.org (S.S.S.); irteja.islam@icddrb.org (I.I.)

**Keywords:** cognitive, psychosocial, crèche, daycare, child development, Bangladesh, Ages and Stages Questionnaire (ASQ), early childhood development (ECD), early childhood care (ECC)

## Abstract

The Developmental Study is part of a larger intervention on “saving of lives from drowning (SoLiD)” where children were enrolled either into crèches (daycare centers) or playpens to prevent drowning in rural Bangladesh. Sampling ~1000 children between the ages of 9–17 months, we compared problem-solving, communication, motor and personal-social outcomes assessed by the Ages and Stages Questionnaire in the two interventions. After controlling for variables such as home stimulation in multivariate regressions, children in crèches performed about a quarter of a standard deviation better in total scores (*p* < 0.10) and 0.45 standard deviations higher in fine motor skills (*p* < 0.05). Moreover, once the sample was stratified by length of exposure to the intervention, then children in crèches performed significantly better in a number of domains: those enrolled the longest (about 5 months) have higher fine motor (1.47, *p* < 0.01), gross motor (0.40, *p* < 0.05) and personal-social skills (0.95, *p* < 0.01) than children in playpens. In addition, children in crèches with the longer exposure (about 5 months) have significantly higher personal-social and problem-solving scores than those in crèches with minimum exposure. Enrollment in crèches of very young children may be positively associated with psychosocial scores after accounting for important confounding variables.

## 1. Introduction

Early stimulation improves developmental outcomes among young children and these outcomes last over long periods [1,2,3]. Interventions aimed at children in their early years also yield greater returns than programs that intervene later [4,5]. Early childhood education and care is thus a policy priority given the potential benefits that early cognitive and other stimulation can have on children’s problem-solving, communication, motor and personal-social outcomes, and the associated economic and social benefits that may arise at a population level [6].

As such programs become more popular, their designs and contributions to cognitive and behavioral outcomes in varied settings are still uncertain. What types of child-focused programmatic activities will have positive psychosocial outcomes in a particular setting? This is an area of active research [7]. Early child care (ECC) programs in low-and-middle-income countries (LMICs) have only been evaluated recently. These programs have tended to include components on improved nutrition, cognitive stimulation, and cash transfers [8,9]. The cognitive stimulation components have entailed home-visitation programs or counseling sessions where the focus is on at-risk children and training of caregivers [1,5,8,10]. High quality studies that describe ECC programs aimed at large populations of children in institutional settings outside the United States are limited [8,9].

More than a third of children between the ages of 3 and 4 years in LMICs are estimated to have either low cognitive or socio-emotional scores, or both [11]. Bangladesh has been very successful in achieving high primary and secondary enrollment rates, with primary school completion doubling in the last 30 years. However, the country does not have a formalized early childhood development plan. As part of its skill development agenda, and to help equalize learning opportunities, Bangladesh is prioritizing a focus on building cognitive and behavioral skills in early childhood via center-based early learning [12].

ECC provided in out-of-home institutional settings (center-based care) is an unusual offering in low-income settings [9]. In Bangladesh, about 23 percent of children 3–5 years old were attending early childhood education in 2009 [13]. In comparison, in Organization for Economic Co-operation and Development (OECD) countries, about 82 percent of children 3–5 years old were enrolled in pre-primary education in 2011 [14,15]. In addition, about one-third of children between 0–2 years in OECD countries were in some kind of formal care, and these rates are increasing [14,15]. Recognizing the importance of early education, national policy is now shifting towards children between 3–5 years of age (referred to as pre-primary school age). While center-based care in a number of settings has shown high returns [5], child outcomes have largely been found to be associated with the quality of day care centers that form part of the ECC programs [16]. In addition to the low coverage of ECC programs in LMIC settings, the quality of most programs is often poor and the cognitive stimulation involved may not be developmentally appropriate, resulting in poor child outcomes [7].

## 2. Background on the SoLiD Program

The Saving of Lives from Drowning (SoLiD) project is one of the largest child drowning prevention projects undertaken in a LMIC. It aimed at enrolling around 80,000 children between 9–36 months in 51 unions (the local geographic administrative boundary) and 7 sub-districts in rural Bangladesh. The SoLiD project included two main interventions to prevent childhood drowning in rural Bangladesh. The first involved the use of playpens as a supervisory aid to prevent children 9–36 months from drowning [17,18]. The second intervention involved the enrollment of children 9–36 months into a crèche during the period of the day when they are most at risk of drowning [17]. The quality of execution of the SoLiD project was high, with fidelity to the initial design, and the outcomes on drowning have been promising [17,18].

Children in crèches under the SoLiD project (referred to as “anchal”, meaning refuge in Bangla) experienced a wide variety of psychosocial stimulation that was provided to them by crèche mothers who underwent a series of trainings and were given a variety of materials for stimulating the children. It is natural, therefore, to investigate how children enrolled in the creche program gained from such inputs, and whether they experienced improvements in psychosocial outcomes. The goal of this paper is to describe the Developmental Study that was designed to investigate children’s psychosocial outcomes and to discuss preliminary results from the baseline survey conducted during 2015. More broadly, the study provides a unique opportunity to examine the early effects of enrollment in crèches among a large group of rural children in a LMIC.

The crèche program under the SoLiD project was designed to be an intensive experience that exposes children (aged 9–36 months and followed up to 48 months) to developmentally appropriate cognitive stimulation. The curriculum was designed by Bangladesh-based child psychologists (to reflect the local context) and included social activities such as story telling, song and dance, and information on personal hygiene and nutrition. Learning included instruction on counting and the rural Bangladesh environment including the geography, flora and fauna. All learning and instruction took place in the local Bangla language. Age-appropriate toys and reading materials based on the local context were also made available.

The crèches operated from 9 a.m. to 1 p.m. six days a week. They were community based, and were run by a crèche mother and her assistant who together supervised 15 to 30 children (a staff–child ratio was about 1:12–1:15). Crèche mothers were trained by master trainers who in turn were trained by the child specialists who designed the curriculum. To ensure local ownership of the crèche, the crèche mother, her assistant, the location of the crèche and other details were decided by a local village committee with support from the project staff. Parent–teacher meetings were regularly organized, and the village committee with support from project staff had oversight over the running of the crèches. Project staff also supervised the activities of the crèche mothers and assistants to assure quality and to provide refresher’s training as needed.

The children in the playpens spent an average of 20 min continuously in the playpen per use, and the playpens were used about three times per day. Hence, the total time spent in the playpens was 60 min per day for seven days in a week. The playpens were most often placed inside the house (84% of cases) and less frequently in the yard (14%). Although no program-based psychosocial stimulations were provided for children in the playpens, caregivers/family members were expected to continue to interact and supervise the children while they were in the playpens. Only one child was allowed in a playpen.

The SoLiD project is thus opportune to examine the influence of an ECC program with a focus on stimulation and early developmental outcomes in a low- and middle-income setting. The project aims to monitor psychosocial outcomes for a sample of children under both the crèche and playpen interventions for at least two years. The objective of this paper is to understand the association between an ECC program with a focus on cognitive stimulation and children’s psychosocial outcomes in rural Bangladesh within a short timeframe—only a handful of rigorous studies looking at this association come from LMICs (and none are from outside Latin America) [9].

## 3. Methods

### 3.1. Design, Participants and Recruitment

This Developmental Study was nested within the larger SoLiD project and involved participants from two (Matlab North and Matlab South) of the seven sub-districts covered in the SoLiD project. Based on a census established for the SoLiD project [17], it identified a total of 7571 children between 9–17 months in March 2015 in these two sub-districts at baseline. Villages were randomly allocated into two areas for the larger SoLiD project: Area 1 had children who received playpens while Area 2 had children being enrolled into the crèches at baseline. The 7571 children across both Areas 1 and 2 were our source population for the developmental study.

In this paper, we describe initial differences that emerged across the two groups. We exploit the design feature that children in both groups experienced varying lengths of exposure as the program was rolled out. Specifically, the difference in dose response is examined, between children randomly enrolled in the playpen (control) and crèche (treatment) groups in relation to how long they were exposed to the interventions.

Eligibility criteria for inclusion in the Developmental Study were that (1) children are between 9–17 months when interviewed; (2) they were enrolled into the SoLiD program on or after 1 January 2015 so that a true baseline is obtained (i.e., exposure to the program was limited); and (3) up to 30 children per village were selected (to prevent an unbalanced design). We calculated a sample size of 1200 (600 in each of Areas 1 and 2) to have a power of 80% to detect a conservative change in psychosocial outcome scores between children under the crèche intervention in Area 2 and those receiving the playpen in Area 1 (this was based on the difference in raw scores and standard deviations observed in other studies in Bangladesh) [19]. We also accounted for the multi-year intervention, correlation between measures over time, and a 20% loss to follow-up while estimating this target sample size. The sample of children was selected using a cluster random sampling technique without replacement. To ensure a total of about 600 children in each arm, we assigned each village a random number in Area 1 and Area 2. We then selected up to 30 children from the villages that were randomly chosen until a total of 600 children was selected from each Area.

### 3.2. Description of Variables

The Ages and Stages Questionnaire (ASQ-3) is a screening tool developed for children between 1 to 66 months of age [20]. It is widely used in the United States to identify potential developmental delays by parents [21]. The version implemented in this study was translated into Bangla and adapted to the local context by a leading expert in child development who was on the team. The adaptations were made to reflect relevant examples for the Bangladeshi context during the assessment while preserving the original questions from the ASQ. Related to five domains, the questions in the ASQ-3 assessed a child’s gross motor, fine motor, communication, problem solving and socioemotional development. The extended version, which consists of 12 questions for each domain, was used; the questions were age-specific and arranged in age bands of two months.

Questions in the ASQ-3 were addressed to parents by trained testers. It took an average of 90 min to conduct an assessment per child. Parents could respond with yes, sometimes, or no (respectively scored as 10, 5 or 0)—12 questions were asked in each domain for a total of 120 points. Table 1 below illustrates each domain with a sample question from the 9-month and 18-month questionnaire. For some questions, the tester actually tested if the child could perform a certain task (indicated by ‘test’). In addition, some questions overlapped and were asked across age categories.

A number of relevant control variables were included in the analyses based on a previous study in Bangladesh which suggests that these variables are important measures of health and wellbeing and are relevant for developmental assessment of children in the Bangladesh context [19]: the child’s gender, age at testing, Family Care Indicators (FCI), household asset index, head circumference, mother’s weight, child’s weight, disability, and exposure. The FCI is a standard set of seven questions around items that the child plays with or interacts with in the home. Each response was coded as yes or no and the total number of responses was added up on a scale of seven (where each ‘yes’ was recorded as one). The questions referred to items that were home-made and bought, but also household materials that were used by the child at home over the last month to play music, color or draw, assist in pretend play, encourage movement, learn shapes, or build blocks. Length of exposure was calculated as time between the date of enrollment of the child into the SoLiD program and the date of interview for the Developmental Study. For the analysis, children were allocated into one of three equal groups based on how much their length of exposure had been (minimum, medium and maximum exposure).

The outcome scores were standardized as z-scores. Unadjusted bivariate associations were first examined between children receiving the playpens and those in crèches for their test scores (both raw and z-scores) and other background characteristics using *t*-test, chi-square and ordinary least square regressions. Ordinary least square regressions were then estimated for each ASQ domain comparing z-scores for children in the crèches with those in the playpens while controlling for relevant characteristics (all control variables were added simultaneously). To account for the sample design, site-specific clustered standard errors (at the village level) and fixed-effect estimates were used to improve estimates. The cluster adjustment was also used to account for the likelihood of children from better off households being located in better neighborhoods/villages, and thus being exposed to better quality teachers, equipment, and lower crèche mother–child ratios within crèches. Sensitivity analyses were done based on length of exposure to the intervention (either crèche or playpen), and the marginal change in outcomes was assessed based on increased exposure.

All testers underwent a two-week training session and inter-observer reliabilities were conducted. Inter-observer reliabilities during data collection using intra-class correlations ranged from 0.93 to 1.0 for different subscales between each of the four testers and the supervisors. In addition, the supervisor conducted (and cross-checked) 10% of the final surveys to ensure comparability and quality control. Participation was voluntary and parents were provided with detailed human consent forms that were explained to them in Bangla. Ethical approval (IRB No. 00004746) for this study was obtained from the Johns Hopkins University and the International Center for Diarrheal Disease Research, Bangladesh Institutional Review Boards.

## 4. Results

### Description of Sample

A total of 1018 children were in the final analytic sample, they were interviewed between April and September 2015 and full data was available for them. Of these, 510 children received just the crèche, while the control group consisted of 508 children who received just the playpen.

Of the original sample of 1435, a total of 417 were not included in the analysis for various reasons. The ASQ score was not accessible for 280 children, socioeconomic measures were not collected for 59 children, and 42 children received both the playpen and crèche—these were all excluded from the analysis. In addition, 21 children came from villages with more than 30 children per village (21 children were thus randomly selected and dropped, and balance across arms was checked to ensure not all children were dropped from one arm). In the final analytic sample, for the treatment group, from Area 2, 80 villages were selected, with village sizes of between 5–80 children. For the control group, from Area 1, 108 villages were selected, with village sizes of between 5–88 children.

The sample was divided into three equal and non-overlapping groups based on length of exposure to the interventions (minimum, average, maximum exposure). The average time between enrollment and interview was found to be 24 days (0–2 months, or “about one month”) for children in the minimum exposure category, and it was 72 days (2–4 months, or “about 2.5 months”) and 156 days (4–8 months, or “about 5 months”) for the average and maximum exposure groups.

In the bivariate analyses, children in the playpen had higher scores than children in the crèche (Table 2). Without adjusting for the control variables, children in crèches had significantly lower scores on all the psychosocial dimensions compared to children receiving playpens. Enrollment in crèches was also associated with significantly lower FCI (at the 1% level) and asset index (at the 10% level). While children in the crèches were not different from children in the playpen in their weight or prevalence of disability, they had slightly lower head circumference (significant at the 5% level) and heavier mothers (significant at 1% level). Children in the crèche also had much lower exposure to the SoLiD intervention (significant at the 1% level). Children in crèches were exposed to crèches for about 52 days on average compared with those in the playpen who have been exposed to playpens for about 115 days (a difference of about two months).

In Table 3 below, we display results of ordinary least squares regressions that control for all the variables shown in Table 2. Separate models were run for each domain of the ASQ test. After controlling for relevant characteristics, children in the crèche performed significantly better in fine motors skills than children in the playpen (Table 3). On other dimensions such as communication, gross motor, personal social and problem solving, there were no differences between the children under the different arms. Child’s age and weight were significantly associated with ASQ scores for selected domains. Younger children, and children with higher weights were associated with better ASQ scores. The FCI was strongly associated with all domain scores and the total scores; a unit increase in FCI was associated with a 0.4 standard deviation increase in scores (significant at 1% level).

In the sensitivity analyses, for the adjusted relationship, longer exposure was associated with larger differences in higher scores obtained for children in the crèches (Table 4). Children in crèches who had been “maximally” exposed (about five months) had a 0.63 standard deviation higher total score (significant at the 5% level) than children in the playpen for a similar length of exposure (Table 4). They also had higher fine motor skills (1.47 standard deviations higher, significant at the 1% level), and better gross motor skills (0.40 standard deviations higher, significant at the 5% level). These children are also reported to have better personal/social skills than children in the playpen who have been enrolled for the same amount of time (0.95 standard deviations, significant at the 1% level). Total scores (in column 6) increase from −0.59 to 0.63, and so do all other scores except fine motor skills that fall from 1.79 to 1.47 standard deviations. The largest difference between these two groups is in problem solving, where it is −1.57 among children with the minimum exposure but −0.07 among children with maximum exposure.

## 5. Discussion

Children in crèches had better total ASQ scores and fine motor skills compared to children in the playpen controlling for relevant characteristics, and the longer that children spent in the crèche, the better they performed with respect to their total ASQ scores, gross motor, fine motor, and personal/social scores.

About 30 developing countries had policies on early childhood development by 2005, however, there are no rigorous studies (apart from a few from Latin America) that have looked at the effect of daycare centers on child development outcomes [5,7,9]. The current study begins to address this evidence gap and evaluates the benefits of early center-based care on child psychosocial outcomes.

Given the differences in FCI, household asset index and head circumference at baseline, it appears that children enrolled in crèches had some initial cognitive *disadvantage* as compared with children enrolled in playpens. However, with exposure to cognitive stimulation under the crèche intervention, children had better psychosocial outcomes if they were in the program longer. For all domains assessed, as children spend more time in the program those in the crèches have higher scores over those in the playpens. The contrast is clearest if we examine children who had the least exposure with those who have had the maximum exposure (panels A and C in Table 4), and this is particularly true for the problem-solving domain.

Out-of-home care for very young children in formal settings in low-middle-income countries such as Bangladesh is an unusual experience. In wealthier countries, such institutions are a common location for early childhood learning. In those settings, it has been established that the *quality* of day care centers is most important in determining psychosocial outcomes [7,22]. In the current study, we do not have measures for quality in the current study. We are aware, however, that extensive efforts were made to ensure that the curriculum was designed based on context-relevant expert guidance and that implementation of the program was of high quality [17]. Related to this concern, it is unclear whether this crèche program can be replicated in other settings. The SoLiD program invested significant resources in ensuring the fidelity to initial design and maintaining the quality of implementation. The study is limited in that current estimates are based on a relatively short time frame. While the lack of data on quality of crèches and the short observation period are important limitations, additional information on these variables would improve external validity but the findings remain robust given these limitations. The study also lacked a pure control group (i.e., those without any intervention). However, it is not expected that the ASQ scores of children in the comparison group (those in the playpens) will be different from those who do not receive any intervention given that no program-based psychosocial stimulations were included for the playpen intervention and children spent limited amount of time (less than one hour) in the playpen per day. The study implements an unusual quasi-experimental design involving a much larger number of children (enrolled in a larger number of crèches) and more robust analyses than many other similar studies. Random treatment assignment and sampling of crèche and playpen villages, and including village fixed effects allows us to control for the unobserved differences across villages (differences in socioeconomic status and culture) that may also account for possibly different background characteristics of children in the crèches. Also, the sample for this study was inflated to accommodate 20% loss to follow-up (which is close to the 19% attrition rate observed due to inability to apply the ASQ assessment).

Further assessment will be needed once the children have had additional exposure to the interventions. It is also possible that parents over (under)-estimate their child’s ability as measured by the ASQ [23]. However, the design feature of having a treatment and control, and also assessments over time will attenuate this problem. There is no reason to believe that parents of children in crèches respond differently from similar parents of children in playpens with respect to underestimation. However, other information biases are likely, given that the ASQ assessment is based on parental report. For instance, parents of children under the crèches may report better performance because they understand that the crèche is a form of preschool that is expected to improve their children’s learning ability. The data collection was conducted without any reference to the interventions, and parents were encouraged to provide objective reports to accurately assess the wellbeing of their children. Moreover, in some studies, parental reports of children’s language abilities or their temperament [3] were closely related to their children’s scores assessed by testers on language and behavior respectively.

## 6. Conclusions

The current study provides a unique opportunity for a rigorous examination of the influence of crèches on children’s psychosocial outcomes in Bangladesh. The results from this assessment indicate that children in the crèche make greater gains over children in the playpen over a short period. Further research is required to understand if these outcomes are sustained, what kinds of children (for example, by gender, socioeconomic status) gain the most, and also what type of teachers or institutional arrangements for daycare settings are most effective. However, this initial work supports the positive impact of crèches, even though these crèches were primarily designed as a drowning prevention strategy.

## Figures and Tables

**Table 1:** Sample questions from ASQ-3 for each domain based on age.

Domain	9 Months	18 Months
Communication	If you call your baby when you are out of sight, does she look in the direction of your voice?	When you ask your child to point to her nose, eyes, hair, feet, ears, and so forth, does she correctly point to at least seven body parts?
Gross motor	Does your baby roll from her back to her tummy, getting both arms out from under her?	Does your child jump with both feet leaving the floor at the same time?
Fine motor	Does your baby reach for small things (the size of a peanut/pea/puffed rice) and touch it with her finger or hand?	Does your child use a turning motion with her hand while trying to turn doorknobs, windup toys, twist tops, or screw lids on and off jars?
Problem solving	If a toy falls from her hand while on her back, if she could see the toy, does she try to reach for it?	After she watches you draw a line from the top of the paper to the bottom with a crayon (or pencil or pen), does your child copy you by drawing a single line on the paper in any direction? (Scribbling back and forth does not count as “yes.”) (Test)
Personal-social	When in front of a large mirror, does your baby reach out to pat the mirror? (Test)	When playing with either a stuffed animal or a doll, does she pretend to rock it, feed it, change its clothes, put it to bed, and so forth?

**Table 2:** Sample characteristics and differences between children in playpen and crèche.

Variables		Playpen (*n* = 508)				Crèche (*n* = 510)				Difference across Interventions? ^1^
		Mean	SD	Min	Max	Mean	SD	Min	Max	
Raw Scores	Communication	78.76	18.82	0	120	76.38	19.74	5	120	**
	Gross motor	88.04	22.67	10	120	84.59	23.37	0	120	**
	Fine motor	74.36	18.95	10	120	71.35	19.7	0	120	**
	Personal/social	72.39	23.5	0	120	67.26	23.95	0	120	***
	Problem solving	78.72	20.31	10	120	73.81	20.32	10	120	***
	Total score	392.27	74.13	50	560	373.4	78.79	30	555	***
Z Scores	Communication	0.07	0.99	−4.19	2.61	−0.08	1	−3.38	2.09	**
	Gross motor	0.07	0.96	−3.62	1.65	−0.08	1.04	−4.64	1.65	**
	Fine motor	0.07	0.98	−4.06	2.46	−0.06	1	−3.74	2.33	**
	Personal/social	0.09	1.01	−2.98	2.43	−0.1	1	−2.98	2.43	***
	Problem solving	0.1	1	−3.26	2.4	−0.11	1	−3.37	2.14	***
	Total score	0.11	0.98	−4.72	2.35	−0.1	1.01	−4.37	2.43	***
Controls	Male	55.12				49.8				
	Age	14.31	2.15	9	18	14.07	2.04	9	18	
	FCI	2.25	1.16	0	7	1.96	1.13	0	7	***
	Asset index	0.05	0.97	−2.72	3.7	−0.05	1.03	−3.56	2.88	
	Head circumference	44.57	1.43	40.1	48.3	44.35	1.4	40	48.4	**
	Mother’s weight	72.73	26.12	19	99.9	79.21	25.5	24.2	99.9	***
	Child's weight	8.85	1.29	5	15	8.76	1.37	5.2	18.5	
	Exposure (days)	115.37	58.92	−131	243	52.49	46.9	−210	188	***
	Disability	8.76	0.5	6	9	8.77	0.54	5	9	

^1^ *** *p* < 0.01, ** *p* < 0.05, Two-sided *t*-tests were conducted for continuous variables and chi-square tests for categorical variables.

**Table 3:** Coefficient estimates from multivariate linear regression models, regressing ASQ z-scores in individual and household characteristics.

Variables	(1)	(2)	(3)	(4)	(5)	(6)
*ASQ Domain:*	Communication	Gross Motor	Fine Motor	Personal/Social	Problem Solving	Total Score
Crèche	0.06	0.27	0.45 **	0.14	−0.06	0.24 *
	(0.22)	(0.22)	(0.17)	(0.16)	(0.16)	(0.13)
Boy	−0.05	0.00	−0.08	−0.04	−0.04	−0.06
	(0.06)	(0.06)	(0.06)	(0.06)	(0.06)	(0.06)
Age	−0.05 ***	−0.08 ***	−0.06 ***	−0.07 ***	−0.07 ***	−0.09 ***
	(0.02)	(0.02)	(0.02)	(0.02)	(0.02)	(0.02)
Family care indicator	0.27 ***	0.22 ***	0.32 ***	0.35 ***	0.33 ***	0.40 ***
	(0.03)	(0.03)	(0.03)	(0.03)	(0.03)	(0.03)
Household asset Index	−0.00	0.02	0.03	0.07 **	0.04	0.04
	(0.04)	(0.04)	(0.04)	(0.03)	(0.04)	(0.04)
Child head circumference	0.01	0.05	0.06 **	0.08 **	0.07 **	0.07 **
	(0.03)	(0.03)	(0.02)	(0.03)	(0.03)	(0.03)
Mother’s weight	−0.00	0.01 **	0.00	0.00	0.00	0.00
	(0.00)	(0.00)	(0.00)	(0.00)	(0.00)	(0.00)
Child’s weight	0.11 ***	0.09 ***	0.08 ***	0.04	0.00	0.08 ***
	(0.04)	(0.04)	(0.03)	(0.03)	(0.03)	(0.03)
Exposure (Days)	0.00	0.00 **	0.00	0.00	−0.00	0.00
	(0.00)	(0.00)	(0.00)	(0.00)	(0.00)	(0.00)
Disability	0.14	0.33 ***	0.07	0.07	0.11	0.20 **
	(0.09)	(0.09)	(0.08)	(0.08)	(0.08)	(0.09)
Constant	−2.98 **	−6.84 ***	−4.50 ***	−4.80 ***	−4.38 ***	−6.48 ***
	(1.26)	(1.55)	(1.22)	(1.41)	(1.12)	(1.30)
Observations	1018	1018	1018	1018	1018	1018
Number of villages	151	151	151	151	151	151
DF	9.00	9.00	9.00	9.00	9.00	9.00
Adjusted R-square	0.13	0.14	0.17	0.16	0.15	0.26
Rho	0.24	0.22	0.30	0.27	0.25	0.24

Notes: *** *p* < 0.01, ** *p* < 0.05, * *p* < 0.10; Robust standard errors in parentheses; Village fixed-effects estimated.

**Table 4:** Coefficient estimate for Crèche intervention from multivariate linear regression models, regressing ASQ z-scores in individual and household characteristics, stratified by length of exposure to intervention.

**Panel A**	**Length of Exposure:**	**Minimum Exposure (About One Month)**					
		**(1)**	**(2)**	**(3)**	**(4)**	**(5)**	**(6)**
	***ASQ Domain:***	**Communication**	**Gross Motor**	**Fine Motor**	**Personal/Social**	**Problem Solving**	**Total Score**
	Crèche	−0.19	−0.40	1.79	−1.37	−1.57 **	−0.59
		(0.98)	(1.40)	(1.20)	(0.87)	(0.74)	(1.25)
	Constant	−4.34	−9.46 ***	−8.74 ***	−4.36 *	−5.68 **	−8.74 ***
		(2.71)	(2.31)	(2.85)	(2.57)	(2.81)	(2.66)
	Observations	340	340	340	340	340	340
	Adjusted R-square	0.14	0.16	0.18	0.18	0.19	0.28
	Rho	0.44	0.46	0.65	0.60	0.57	0.44
**Panel B**	**Length of exposure:**	**Medium Exposure (About 2.5 months)**					
	Crèche	−0.90***	0.24	1.11	0.54	1.04 ***	0.60
		(0.23)	(0.24)	(0.88)	(1.37)	(0.23)	(0.62)
	Constant	−2.94	−4.75 *	−3.61 *	−5.30 **	−7.75 ***	−6.82 ***
		(1.94)	(2.68)	(2.06)	(2.47)	(1.75)	(2.19)
	Observations	341	341	341	341	341	341
	Adjusted R-square	0.19	0.16	0.20	0.20	0.21	0.30
	Rho	0.43	0.26	0.38	0.39	0.50	0.28
**Panel C**	**Length of exposure:**	**Maximum Exposure (About 5 months)**					
	Crèche	−0.41	0.40 **	1.47 ***	0.95 ***	−0.07	0.63 **
		(0.34)	(0.18)	(0.18)	(0.34)	(0.17)	(0.24)
	Constant	−2.01	−6.44 **	−4.12 *	−6.74 ***	−0.85	−5.50 ***
		(2.14)	(2.71)	(2.17)	(2.39)	(2.11)	(1.85)
	Observations	337	337	337	337	337	337
	Adjusted R-square	0.10	0.13	0.18	0.17	0.13	0.25
	Rho	0.32	0.31	0.53	0.38	0.31	0.35
**Crèche means significantly different across Minimum and Maximum exposure?**		**No**	**No**	**No**	**Yes**	**Yes**	**No**

Notes: *** *p* < 0.01, ** *p* < 0.05, * *p* < 0.10; Robust standard errors in parentheses; Village fixed-effects estimated; All variables in Table 1 are included in the regressions.

## Abbreviations

The following abbreviations are used in this manuscript:

ASQ	Ages and Stages Questionnaire
ECC	Early Childhood Care
ECD	Early Childhood Development
FCI	Family Care Indicators
LMICs	Low and middle-income countries
OECD	Organization for Economic Co-operation and Development
SoLiD	Saving of Lives from Drowning
